# Persistence of Monoclinic Crystal Structure in 3D Second‐Order Topological Insulator Candidate 1*T*′‐MoTe_2_ Thin Flake Without Structural Phase Transition

**DOI:** 10.1002/advs.202101532

**Published:** 2021-12-19

**Authors:** Bo Su, Yuan Huang, Yan Hui Hou, Jiawei Li, Rong Yang, Yongchang Ma, Yang Yang, Guangyu Zhang, Xingjiang Zhou, Jianlin Luo, Zhi‐Guo Chen

**Affiliations:** ^1^ Beijing National Laboratory for Condensed Matter Physics Institute of Physics Chinese Academy of Sciences Beijing 100190 China; ^2^ School of Physical Sciences University of Chinese Academy of Sciences Beijing 100190 China; ^3^ Songshan Lake Materials Laboratory Dongguan 523808 China; ^4^ School of Materials Science and Engineering Tianjin University of Technology Tianjin 300384 China; ^5^ Collaborative Innovation Center of Quantum Matter Beijing China

**Keywords:** 1*T*′‐MoTe_2_, Raman spectroscopy, second‐order topological insulator, structural phase transition, van der Waals materials

## Abstract

A van der Waals material, MoTe_2_ with a monoclinic 1*T*′ crystal structure is a candidate for 3D second‐order topological insulators (SOTIs) hosting gapless hinge states and insulating surface states. However, due to the temperature‐induced structural phase transition, the monoclinic 1*T*′ structure of MoTe_2_ is transformed into the orthorhombic *T*_d_ structure as the temperature is lowered, which hinders the experimental verification and electronic applications of the predicted SOTI state at low temperatures. Here, systematic Raman spectroscopy studies of the exfoliated MoTe_2_ thin flakes with variable thicknesses at different temperatures, are presented. As a spectroscopic signature of the orthorhombic *T*_d_ structure of MoTe_2_, the out‐of‐plane vibration mode *D* at ≈ 125 cm^–1^ is always visible below a certain temperature in the multilayer flakes thicker than ≈ 27.7 nm, but vanishes in the temperature range from 80 to 320 K when the flake thickness becomes lower than ≈ 19.5 nm. The absence of the out‐of‐plane vibration mode *D* in the Raman spectra here demonstrates not only the disappearance of the monoclinic‐to‐orthorhombic phase transition but also the persistence of the monoclinic 1*T*′ structure in the MoTe_2_ thin flakes thinner than ≈ 19.5 nm at low temperatures down to 80 K, which may be caused by the high enough density of the holes introduced during the gold‐enhanced exfoliation process and exposure to air. The MoTe_2_ thin flakes with the low‐temperature monoclinic 1*T*′ structure provide a material platform for realizing SOTI states in van der Waals materials at low temperatures, which paves the way for developing a new generation of electronic devices based on SOTIs.

## Graphical abstract

The transition metal dichalcogenide MoTe_2_ thin flakes thinner than ≈ 19.5 nm do not experience a monoclinic‐to‐orthorhombic phase transition and still have a monoclinic 1*T*′ structure at temperatures down to 80 K, which provides a material platform for realizing 3D second‐order topological insulator states in van der Waals materials at low temperatures.

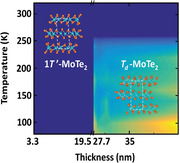

## 1 Introduction

**Figure 1:**
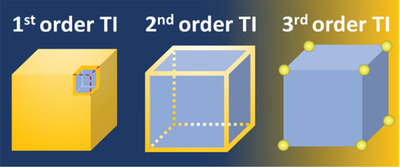
Schematic of the topologically protected states in 3D topological insulators (TIs). The first‐, second‐, and third‐order 3D TIs have the topologically protected states (shown in bright yellow) on their 2D surfaces (left panel), 1D hinges (middle panel), and 0D corners (right panel), respectively.

A topological insulator (TI), which is a type of quantum material with time‐reversal‐symmetry‐protected gapless states on the surfaces or along the edges and insulating states in the bulk, has attracted intensive attention due to its novel physical properties and potential applications in spintronic devices, terahertz detectors, and quantum computing.^[^ ^1^, ^2^, ^3^, ^4^, ^5^, ^6^, ^7^, ^8^ ^]^ Lately, the concept of topological insulator was extended to a new class of systems called higher‐order topological insulators (HOTIs): *d*‐dimensional *n*th‐order TIs have symmetry protected gapless (*d–n*)‐dimensional boundary states (see **Figure** **1**). Therein, intrinsic 3D second‐order topological insulators (SOTIs) exhibit topologically protected gapless states on the 1D hinges, but host insulating states on the 2D surfaces and in the 3D bulk.^[^ ^9^, ^10^, ^11^, ^12^, ^13^, ^14^, ^15^, ^16^, ^17^ ^]^ Furthermore, it was suggested that the topologically protected 1D hinge state of 3D SOTIs sheds new light on the development of novel electronic applications based on Majorana bound states and surface quantum anomalous Hall effect, such as topological quantum computers and chiral circuit interconnects.^[^ ^18^, ^19^ ^]^ However to date, few realistic materials have been identified experimentally as 3D SOTIs.^[^ ^18^, ^20^ ^]^ A natural question to ask is whether a broad class of 3D SOTIs can be found experimentally.

Very recently, a transition metal dichalcogenide MoTe_2_ with the monoclinic crystal structure (i.e., 1*T*′ phase with centrosymmetric space group *P*2_1_/*m*) was theoretically predicted to be a candidate for 3D SOTIs.^[^ ^21^, ^22^ ^]^ However, when the bulk crystal of 1*T*′‐MoTe_2_ is cooled below temperature *T* ≈ 250 K, its monoclinic structure would transition into the orthorhombic structure (i.e., *T*_d_ phase with non‐centrosymmetric space group *Pmn*2_1_) which was revealed to exhibit a type‐II Weyl semimetal (WSM) ground state.^[^ ^23^, ^24^, ^25^, ^26^, ^27^, ^28^, ^29^, ^30^, ^31^ ^]^ Therefore, accompanied with the temperature‐induced structural phase transition from high‐temperature 1*T*′ to low‐temperature *T*_d_ phase, an electronic topological phase transition from a SOTI to a WSM is expected to take place in the bulk MoTe_2_ crystal, that is, the SOTI state in 1*T*′‐MoTe_2_ would be destroyed at low temperatures. The occurrence of the structural (or topological) phase transition around *T* ≈ 250 K in the bulk crystal of 1*T*′‐MoTe_2_ means that (i) it is challenging to observe the predicted SOTI state in 1*T*′‐MoTe_2_ because of the high‐temperature‐induced blurring of the energy dispersions of its gapless hinge states, and (ii) it is difficult to make use of the SOTI state in 1*T*′‐MoTe_2_ to design a new generation of electronic devices working stably at low temperatures. Therefore, it is significant for the experimental identification and the electronic application of the SOTI state in 1*T*′‐MoTe_2_ to maintain the monoclinic crystal structure at low temperatures. Up till now, the monoclinic 1*T*′ crystal structure of MoTe_2_ at low temperatures was mainly realized by applying ultrashort laser pulses, exerting mechanical pressure, or doping carriers.^[^ ^32^, ^33^, ^34^ ^]^ It is worth noticing that the bulk crystal of 1*T*′‐MoTe_2_ can be mechanically exfoliated into thin flakes because the bonding between MoTe_2_ layers is van der Waals type. Nevertheless, whether the MoTe_2_ thin flakes can still have the monoclinic 1*T*′ crystal structure at low temperatures remains elusive.

## 2 Results

**Figure 2:**
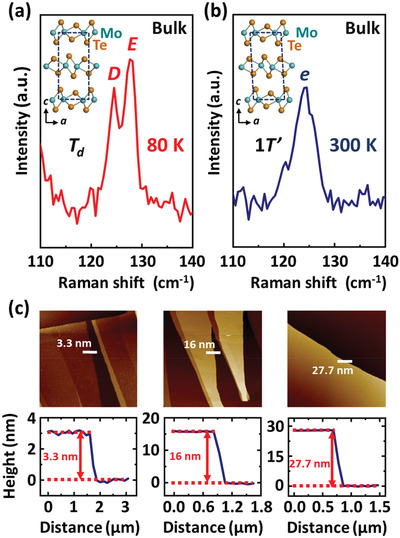
a) The two vibration modes *D* and *E* in the Raman spectrum of the MoTe_2_ bulk crystal with the orthorhombic *T*_d_ structure measured at *T* = 80 K. b) The vibration mode *e* in the Raman spectrum of the MoTe_2_ bulk crystal with the monoclinic 1*T*′ structure at *T* = 300 K. The up left insets in (a, b) show the *T*_d_ and 1*T*′ structure of MoTe_2_, respectively. c) Upper panels: atomic‐force‐microscopy images of the MoTe_2_ flakes. Lower panels: flake thicknesses along the white lines on the upper panels.

Raman spectroscopy is an experimental technique which is directly sensitive to the crystal structure.^[^ ^35^, ^36^, ^37^, ^38^, ^39^, ^40^, ^41^, ^42^, ^43^, ^44^, ^45^, ^46^, ^47^, ^48^, ^49^, ^50^, ^51^, ^52^ ^]^ Previous Raman spectroscopy studies of the bulk 1*T*′‐MoTe_2_ crystals indicate that in the high‐temperature 1*T*′ phase at *T* ≥ 250 K upon cooling (or at *T* ≥ 260 K upon warming), the out‐of‐plane vibration mode *D* around 125 cm^–1^ is Raman in‐active (i.e., only infrared active) and is absent in the Raman spectra due to the centrosymmetry of the monoclinic 1*T*′ structure, while in the low‐temperature *T*_d_ phase at *T* < 250 K upon cooling (or at *T* < 260 K upon warming), the out‐of‐plane vibration mode *D* becomes both Raman‐ and infrared‐active and can be probed by Raman spectroscopy owing to the centrosymmetry breaking in the orthorhombic *T*_d_ structure (see the two vibration modes *D* and *E* in the Raman spectra of the MoTe_2_ bulk crystal with the orthorhombic *T*_d_ structure measured at *T* = 80 K in **Figure** **2**a, the vibration mode *e* in the Raman spectra of the MoTe_2_ bulk crystal with the monoclinic 1*T*′ structure at *T* = 300 K in Figure 2b, and the Raman spectra of the MoTe_2_ bulk crystal in the energy range from 60 to 300 cm^–1^ in Figure S1, Supporting Information).^[^ ^35^, ^36^, ^37^, ^38^, ^39^, ^40^ ^]^ Thus, the presence of the out‐of‐plane vibration mode *D* can be regarded as a spectroscopic signature of the temperature‐driven structural phase transition in MoTe_2_ from the high‐temperature monoclinic 1*T*′ structure to the low‐temperature orthorhombic *T*_d_ structure. Raman spectroscopy investigations of the thickness dependence and temperature evolution of the out‐of‐plane vibration mode *D* enables us to gain insights into the fate of the monoclinic‐to‐orthorhombic phase transition in the MoTe_2_ thin flakes at low temperatures.

**Figure 3:**
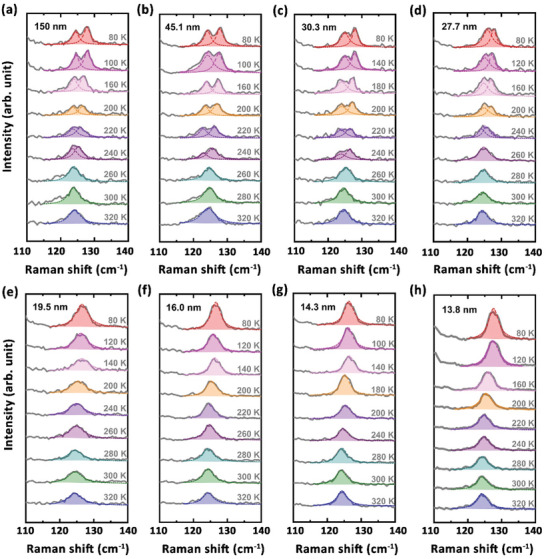
Thickness evolution of the representative Raman spectra of the MoTe_2_ thin flakes measured in the temperature range from 80 to 320 K. The dashed colored curves show the Lorentzian fits to the peak‐like features.

In order to obtain the MoTe_2_ thin flakes, we exfoliated the 1*T*′‐MoTe_2_ bulk crystals grown by chemical vapor‐transport method using iodine as the transport agent.^[^ ^32^ ^]^ The MoTe_2_ thin flakes were fabricated using the gold‐enhanced exfoliation method.^[^ ^41^ ^]^ Figure 2c displays three typical thicknesses of the exfoliated MoTe_2_ flakes characterized by atomic force microscopy. Then, we used a 532 nm laser to measure the Raman spectra of the MoTe_2_ thin flakes at different temperatures in the parallel‐polarized configuration (i.e., the electrical field of the linearly polarized incident light is parallel to the electrical field of the linearly polarized scattered light, here). **Figure** **3** depicts the thickness evolution of the representative Raman spectra of the exfoliated MoTe_2_ thin flakes measured in the temperature range from 80 to 320 K (see the representative Raman spectra plotted in the energy range from 60 to 300 cm^–1^ in Figure S2, Supporting Information, and the Raman spectra of the MoTe_2_ thin flakes with the thicknesses of ≈ 31.1 nm and ≈ 28.5 nm in Figure S3a, b, Supporting Information). For the MoTe_2_ flakes thicker than ≈ 27.7 nm, the out‐of‐plane vibration mode *D* around 125 cm^–1^ is invisible in their Raman spectra at *T* ≥ 260 K, but the mode *D*, together with the mode *E* around 128 cm^–1^, is present in their Raman spectra measured at *T* < 260 K (see Figure 3a–c), which indicates that the monoclinic‐to‐orthorhombic phase transition occurs in the MoTe_2_ flakes thicker than ≈ 27.7 nm. It is worth noticing that for the MoTe_2_ thin flakes thinner than ≈ 19.5 nm, a mode is present around 128 cm^–1^ in the Raman spectra measured in the temperature range from 80 to 320 K, while the mode *D*, which should appear around 125 cm^–1^ in the Raman spectra of the MoTe_2_ crystals with the orthorhombic *T*_d_ structure, not only is invisible at *T* ≥ 260 K, but also disappears at low temperatures down to 80 K (see Figure 3d–f), revealing the vanishing of the monoclinic‐to‐orthorhombic phase transition and the persistence of the monoclinic 1*T*′ structure at temperatures above 80 K in the MoTe_2_ thin flakes thinner than ≈ 19.5 nm.

**Figure 4:**
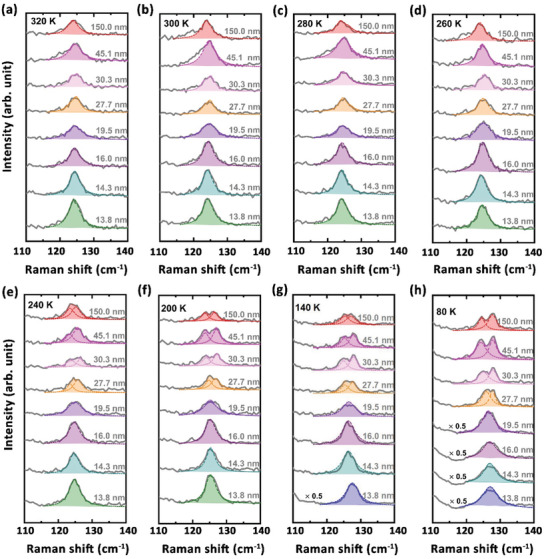
Temperature dependence of the representative Raman spectra of the MoTe_2_ thin flakes with the thickness varying from ≈ 150.0 to ≈ 13.8 nm. The dashed colored curves show the Lorentzian fits to the peak‐like features. The Raman spectra labeled with “× 0.5” in (e, f) are shown with the half of the intensities.

To check the thickness dependence of the monoclinic‐to‐orthorhombic transition in the MoTe_2_ flakes, we plotted the temperature dependence of the representative Raman spectra of the exfoliated MoTe_2_ thin flakes with the thickness varying from ≈ 150 to ≈ 13.8 nm in **Figure** **4** (see the Raman spectra of the MoTe_2_ thin flakes measured at *T* = 180, 160, 120, and 100 K in Figure S3c–f, Supporting Information). When 260 K ≤ *T* ≤ 320 K, the mode *D* is always invisible in the Raman spectra of the MoTe_2_ flakes with the thickness ranging from ≈ 150 to ≈ 13.8 nm (see Figure 4a–c), which is in agreement with the presence of the monoclinic 1*T*′ structure in the MoTe_2_ bulk crystals at *T* ≥ 260 K upon warming. By contrast, when 80 K ≤ *T* < 260 K, the mode *D* is visible in the Raman spectra of the MoTe_2_ flakes thicker than ≈ 27.7 nm, and then becomes absent in the Raman spectra of the MoTe_2_ thin flakes thinner than ≈ 19.5 nm (see Figure 4d–f), which also manifests the disappearance of the monoclinic‐to‐orthorhombic phase transition above 80 K and the existence of the low‐temperature monoclinic 1*T*′ structure in the MoTe_2_ thin flakes thinner than ≈ 19.5 nm.

**Figure 5:**
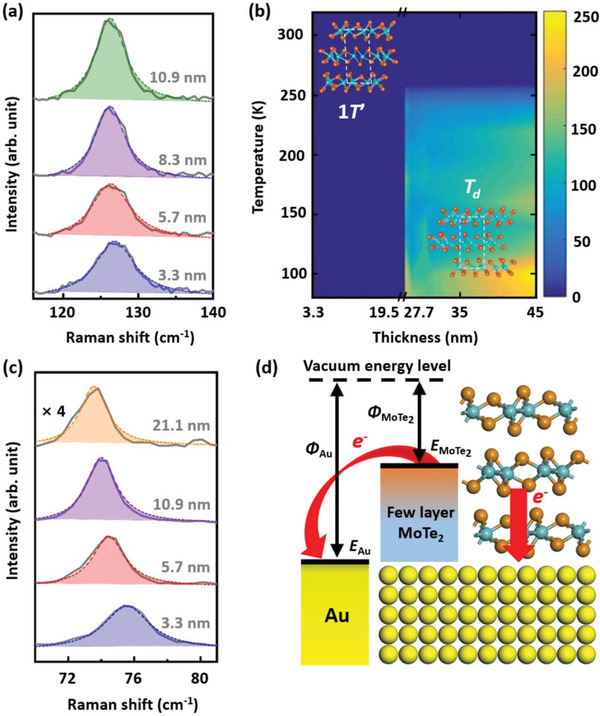
a) Raman spectra of the MoTe_2_ ultrathin flakes with the thicknesses of ≈ 3.3, ≈ 5.7, ≈ 8.3, and ≈ 10.9 nm measured at *T* = 80 K. The dashed curves show the Lorentzian fits to the peaks. b) Color scale map of the mode *D* intensities obtained by the Lorentzian fits as a function of thickness and temperature. c) Out‐of‐plane vibration mode ≈ 74 cm^−1^ in the Raman spectra of the MoTe_2_ ultrathin flakes with the thicknesses of ≈ 3.3, ≈ 5.7, ≈ 10.9, and ≈ 21.1 nm measured at *T* = 80 K. The dashed curves show the Lorentzian fits to the peaks. The vibration mode labeled with“× 4”in (c) is displayed with the fourfold intensity. d) Schematic of the transfer of electrons from the MoTe_2_ thin flake to the golden substrate in the exfoliating process. Here, *ϕ*_MoTe2_ and *ϕ*_Au_ represent the work functions of MoTe_2_ and gold, respectively.

To further confirm the existence of the low‐temperature monoclinic crystal structure in the MoTe_2_ thin flakes, we measured the Raman spectra of the MoTe_2_ ultrathin flakes with the thicknesses lower than those shown in Figures 3 and 4 at *T* = 80 K. As displayed in **Figure** **5**a, a mode is present around 128 cm^–1^, but the mode *D*, which should be visible around 125 cm^–1^ in the Raman spectra of the MoTe_2_ crystals with the orthorhombic *T*_d_ structure, is absent in the Raman spectra of the MoTe_2_ ultrathin flakes with several representative thicknesses of ≈ 10.9, ≈ 8.3, ≈ 5.7,  and ≈ 3.3 nm, which indicates the persistence of the monoclinic 1*T*′ structure in the MoTe_2_ ultrathin flakes with the thicknesses down to ≈ 3.3 nm at *T* = 80 K. In Figure 5b, we plotted the false‐color map of the intensities of the mode *D* obtained by the Lorentzian fits as a function of thickness and temperature. As the dark‐blue color in Figure 5b represents the absence of the mode *D* in the Raman spectra of the MoTe_2_ thin flakes with the monoclinic 1*T*′ structure, while the bright colors, such as yellow and green, show the presence of the mode *D* in the Raman spectra of the MoTe_2_ thin flakes with the orthorhombic *T_d_* structure, the false‐color map of the intensity of the mode *D* in Figure 5b can be regarded as the structural‐phase diagram of the MoTe_2_ flakes as a function of thickness and temperature.

## 3 Discussion

A previously reported theoretical investigation of the structural phase transition in MoTe_2_ indicates that (i) across the monoclinic‐to‐orthorhombic phase transition in the bulk MoTe_2_ crystals, a net charge is transferred from the intralayer bonding state around the *Y* point of the Brillouin zone to the interlayer antibonding states along the Γ–*A* direction near Fermi energy, (ii) the net charge transfer lowers the total energy of the system, which can induce the occurrence of the monoclinic‐to‐orthorhombic phase transition in the bulk MoTe_2_ crystals, (iii) the energy difference between the monoclinic phase and the orthorhombic phase is quite small (i.e., ≈ 0.4 meV per unit cell for MoTe_2_), (iv) the monoclinic (or orthorhombic) structure of the bulk MoTe_2_ crystals can be stabilized by the hole (or electron) doping.^[^ ^53^ ^]^ Therefore, the doping type and the doping level at room temperature are expected to induce different behaviors of the structural phase transition in MoTe_2_, which can be supported by the observation of the different crystal structures of the thin flakes with the same thickness and the existence of the intermediate phase corresponding to neither the monoclinic phase nor the orthorhombic phase.^[^ ^43^, ^44^, ^45^, ^46^, ^47^, ^48^ ^]^ It is worth noticing that the phonon energies of the transition metal dichalcogenides can show significant dependence on the doping level and the doping type.^[^ ^49^ ^]^ For 2*H*‐MoTe_2_, the out‐of‐plane vibration mode A_1g_ exhibits a blue (or red) shift with the enhancement of the hole (or electron) concentration, while the A_1g_ mode shows a red shift with the decrease in the flake thickness.^[^ ^50^ ^]^ For the MoTe_2_ flakes here, the out‐of‐plane vibration mode around 74 cm^–1^ in Figure 5c displays a blue shift of ≈ 2 cm^–1^ as the flake thickness decreases from 21.1 to 3.3 nm, which implies that the hole concentration within the MoTe_2_ flakes increases with the decrease in the flake thickness. Moreover, considering that (i) the MoTe_2_ thin flakes in our manuscript were fabricated using the gold‐enhanced exfoliation method (see ref. ^[^ ^41^ ^]^ or the Experimental Section in our manuscript), and (ii) the work function of gold is larger than that of MoTe_2_,^[^ ^51^, ^52^ ^]^ electrons are expected to be transferred from our MoTe_2_ thin flakes to the golden substrate in the process of exfoliating the crystals (see the schematic in Figure 5d), which can lead to the hole doping in our MoTe_2_ thin flakes. In addition, an exposure of our MoTe_2_ thin flakes to air can also result in the hole doping.^[^ ^54^, ^55^, ^56^, ^57^, ^58^ ^]^ Therefore, we speculate that (i) when the MoTe_2_ flakes, which are fabricated using the gold‐enhanced exfoliation method and are exposed to air, become thin enough (i.e., the flake thickness here is lower than ≈ 19.5 nm), the hole concentration within the MoTe_2_ flakes is likely to be high enough to stabilize the monoclinic structure (i.e., the monoclinic‐to‐orthorhombic phase transition is absent in the MoTe_2_ flakes thinner than ≈ 19.5 nm at least above 80 K), and that (ii) when the MoTe_2_ flakes fabricated by the gold‐enhanced exfoliation method are thick enough (i.e., the flake thickness here is larger than ≈ 27.7 nm), the hole concentration within the MoTe_2_ flakes may be not high enough so that the monoclinic structure of the MoTe_2_ flakes thicker than ≈ 27.7 nm would transition into the orthorhombic structure (i.e., the monoclinic‐to‐orthorhombic phase transition can take place in the MoTe_2_ flakes thicker than ≈ 27.7 nm). In brief, the possible reason why the MoTe_2_ flake thickness is special for the structural phase transition is that as the flake thickness decreases, the concentration of the holes introduced during the gold‐enhanced exfoliation process and exposure to air is expected to become higher and higher so that the monoclinic structure of the MoTe_2_ thin flake with the thickness below a critical value can be stable at low temperatures.

It is worth noticing that in the process of decreasing the MoTe_2_ flake thickness by the gold‐enhanced exfoliation method, the increase in the hole concentration (i.e., *n*) within the flakes may be accompanied with the enhancement of the local Coulomb repulsion *U* since the average distance (i.e., *r*) between two charges becomes smaller. It was reported that the local Coulomb repulsion *U* has an effect of stabilizing the orthorhombic crystal structure, while the monoclinic crystal structure can be stabilized by the hole doping (see Figure 3 of ref. ^[^ ^53^ ^]^). Therefore, the competition between the hole doping and the local Coulomb repulsion should play a significant role in determining whether the MoTe_2_ flake goes into the monoclinic structure or the orthorhombic structure. Since (i) the local Coulomb repulsion *U* can be regarded to be approximately proportional to the inverse (i.e., 1/*r*) of the average distance between two charges, and (ii) the hole concentration (i.e., *n*) of the MoTe_2_ flake is approximately linear with the inverse (1/*V*) of the flake volume (*V* ∝*r* ^3^) and is also proportional to the inverse (1/*r* ^3^) of the *r* cubed, the local Coulomb repulsion *U* can be roughly deemed to be proportional to the hole concentration to the one‐third power, that is, *U* ∝ *n* ^1/3^. The approximately linear relationship between *U* and *n* ^1/3^ means that (i) when the concentration (i.e., *n*–*n*_0_) of the doped holes increases (due to a decrease in the flake thickness) but is not high enough (i.e., (*n*–*n*_0_)/*n*_0_ << 1, here *n*_0_ is the hole concentration before doping), the local Coulomb repulsion *U* would increase so sharply that the effect of the local Coulomb repulsion *U* on the crystal structure can be comparable to that of the hole doping, which may destroy the hole‐doping‐induced stabilization of the monoclinic crystal structure and thus, may result in the weak temperature dependence of the structural transition temperature of the MoTe_2_ flakes thicker than 27.7 nm; and (ii) when the concentration of the doped holes continues to increase (owing to the continuous decrease in the flake thickness), the growth velocity of the local Coulomb repulsion *U* would decrease notably and then would be lower than the constant growth velocity of the concentration of the doped holes, which may ultimately make the effect of the hole doping on the crystal structure have an advantage over that of the local Coulomb repulsion *U* and therefore may lead to the abrupt lowering of the structural‐phase‐transition temperature in the MoTe_2_ flakes with the thicknesses ranging from 19.5 to 27.7 nm.

## 4 Conclusion

In summary, using Raman spectroscopy, we have investigated the crystal structures of the exfoliated 1*T*′‐MoTe_2_ thin flakes with different thicknesses as a function of temperature. When the MoTe_2_ flake thicknesses are larger than ≈ 27.7 nm, the out‐of‐plane vibration mode *D* appears around 125 cm^–1^ in the Raman spectra at *T* < 260 K and then becomes invisible at *T* ≥ 260 K, which indicates that the MoTe_2_ flakes thicker than ≈ 27.7 nm undergo the structural phase transition from the monoclinic 1*T*′ structure to the orthorhombic *T*_d_ structure. When the MoTe_2_ flake thicknesses are thinner than ≈ 19.5 nm, the mode *D* is always absent in the Raman spectra even though the temperature is increased from 80 to 320 K, which demonstrates the absence of a temperature‐induced structural phase transition and the retention of the monoclinic 1*T*′ structure at low temperatures down to 80 K in the MoTe_2_ flakes thinner than ≈ 19.5 nm. The possible reason for the persistence of the monoclinic 1*T*′ structure at low temperatures is that as the MoTe_2_ flake thickness is decreased below a critical value, the concentration of the holes introduced during the gold‐enhanced exfoliation process and exposure to air is expected to become high enough to stabilize the monoclinic structure. Our work not only constitutes a significant step toward the experimental realization of the predicted SOTI state in the van der Waals material 1*T*′‐MoTe_2_ but also paves the way for the quest of SOTI states in realistic materials at low temperatures.

## 5 Experimental Section

### Synthesis and Exfoliation of the 1T'‐MoTe_2_ Bulk Crystals

The 1*T*′‐MoTe_2_ single crystals were prepared by the chemical vapor transport method.^[^ ^32^ ^]^ First, the raw materials of the polycrystalline MoTe_2_ were prepared. The Mo and Te powders with a chemical stoichiometric ratio of 1:2 were first mixed and pressed into pellets. The pellets were then sealed in an evacuated quartz tube and heated to 800 ℃ within 20 h. After annealing for 7 days, the tube was quenched into ice water quickly. At last, the prepared precursor and transport agent I_2_ were sealed in an evacuated quartz tube and placed in a two‐zone tube furnace with a temperature gradient from 1000 ℃ to 900 ℃, and that temperature gradient was kept for 7 days. At the end of the sequence, this quartz tube was quenched in the ice water. The 1*T*′‐MoTe_2_ thin flakes were fabricated using the gold‐enhanced exfoliation method.^[^ ^41^ ^]^ First, a thin adhesion metal layer of Ti was evaporated on the Si/SiO_2_ substrate using an electron evaporation system (Peva‐600E), then a thin Au layer was deposited onto the pre‐prepared metal layer of Ti. After that, the 1*T*′‐MoTe_2_ single crystals were mechanically exfoliated using the white tape (3 M scotch) and were cleaved several times along the *c*‐axis. Lastly, the freshly cleaved layered 1*T*′‐MoTe_2_ crystals together with the 3 M scotch tape were put onto the processed substrate and pressed vertically using a gentle pressure for ≈ 1 min, then the tape was peeled off from the substrate

### Polarized Raman Measurements

The Raman spectroscopy measurements were carried out on a HORIBA LabRAM HR Evolution Raman spectrometer using a 532 nm laser. The spectroscopy was acquired in a backscattering geometry with the configurations of the incident and scattered photons polarized parallel to each other. For the Raman measurements in the temperature range from 80 to 320 K, the sample temperature was controlled using a nitrogen cooled Linkam TS600 hot stage.

## Conflict of Interest

The authors declare no conflict of interest.

## Author Contributions

B.S. and Y.H. contributed equally to this work. Project concept and supervision: Z.‐G.C. Carrying out of Raman experiments: B.S. and Y.H.H. Exfoliation of bulk crystals into thin flakes: Y.H. AFM measurements: J.L., R.Y., and B.S. Data analysis: Z.‐G.C., B.S., Y.Y., Y.M., G.Z., X.Z., and J.L. Writing of the manuscript: Z.‐G.C. and B.S.

## Supporting information

Supporting Information

## Data Availability

The data that support the findings of this study are available from the corresponding author upon reasonable request.

## References

[1] C. L. Kane , E. J. Mele , Phys. Rev. Lett. 2005, 95, 146802. 16241681 10.1103/PhysRevLett.95.146802

[2] B. A. Bernevig , T. L. Hughes , S. C. Zhang , Science 2006, 314, 1757. 17170299 10.1126/science.1133734

[3] M. König , S. Wiedmann , C. Brüne , A. Roth , H. Buhmann , L. W. Molenkamp , X.‐L. Qi , S.‐C. Zhang , Science 2007, 318, 766. 17885096 10.1126/science.1148047

[4] L. Fu , C. L. Kane , E. J. Mele , Phys. Rev. Lett. 2007, 98, 106803. 17358555 10.1103/PhysRevLett.98.106803

[5] J. E. Moore , L. Balents , Phys. Rev. B 2007, 75, 121306.

[6] H. J. Zhang , C.‐X. Liu , X.‐L. Qi , X. Dai , Z. Fang , S.‐C. Zhang , Nat. Phys. 2009, 5, 438.

[7] D. Hsieh , D. Qian , L. Wray , Y. Xia , Y. S. Hor , R. J. Cava , M. Z. Hasan , Nature 2008, 452, 970. 18432240 10.1038/nature06843

[8] Y. L. Chen , J. G. Analytis , J. H. Chu , Z. K. Liu , S. K. Mo , X. L. Qi , H. J. Zhang , D. H. Lu , X. Dai , Z. Fang , S. C. Zhang , I. R. Fisher , Z. Hussain , Z. X. Shen , Science 2009, 325, 178. 19520912 10.1126/science.1173034

[9] W. A. Benalcazar , B. A. Bernevig , T. L. Hughes , Science 2017, 357, 61. 28684520 10.1126/science.aah6442

[10] W. A. Benalcazar , B. A. Bernevig , T. L. Hughes , Phys. Rev. B 2017, 96, 245115.

[11] Z. Song , Z. Fang , C. Fang , Phys. Rev. Lett. 2017, 119, 246402. 29286745 10.1103/PhysRevLett.119.246402

[12] J. Langbehn , Y. Peng , L. Trifunovic , F. V. Oppen , P. W. Brouwer , Phys. Rev. Lett. 2017, 119, 246401. 29286744 10.1103/PhysRevLett.119.246401

[13] F. Schindler , A. M. Cook , M. G. Vergniory , Z. Wang , S. S. P. Parkin , B. A. Bernevig , T. Neupert , Sci. Adv. 2018, 4, eaat0346. 29869644 10.1126/sciadv.aat0346 PMC5983919

[14] M. Ezawa , Phys. Rev. Lett. 2018, 120, 026801. 29376716 10.1103/PhysRevLett.120.026801

[15] B.‐Y. Xie , H.‐F. Wang , H.‐X. Wang , X.‐Y. Zhu , J.‐H. Jiang , M.‐H. Lu , Y.‐F. Chen , Phys. Rev. B 2018, 98, 205147.

[16] H. Xue , Y. Yang , F. Gao , Y. Chong , B. Zhang , Nat. Mater. 2019, 18, 108. 30598539 10.1038/s41563-018-0251-x

[17] X.‐L. Sheng , C. Chen , H. Liu , Z. Chen , Z.‐M. Yu , Y. X. Zhao , S. A. Yang , Phys. Rev. Lett. 2019, 123, 256402. 31922761 10.1103/PhysRevLett.123.256402

[18] F. Schindler , Z. Wang , M. G. Vergniory , A. M. Cook , A. Murani , S. Sengupta , A. Y. Kasumov , R. Deblock , S. Jeon , I. Drozdov , H. Bouchiat , S. Guéron , A. Yazdani , B. A. Bernevig , T. Neupert , Nat. Phys. 2018, 14, 918. 30349581 10.1038/s41567-018-0224-7 PMC6195185

[19] Y.‐F. Zhao , R. Zhang , R. Mei , L.‐J. Zhou , H. Yi , Y.‐Q. Zhang , J. Yu , R. Xiao , K. Wang , N. Samarth , M. H. W. Chan , C.‐X. Liu , C.‐Z. Chang , Nature 2020, 588, 419. 33328665 10.1038/s41586-020-3020-3

[20] Y.‐B. Choi , Y. Xie , C.‐Z. Chen , J. Park , S.‐B. Song , J. Yoon , B. J. Kim , T. Taniguchi , K. Watanabe , J. Kim , K. C. Fong , M. N. Ali , K. T. Law , G.‐H. Lee , Nat. Mater. 2020, 19, 974. 32632280 10.1038/s41563-020-0721-9

[21] F. Tang , H. C. Po , A. Vishwanath , X. G. Wan , Nat. Phys. 2019, 15, 470.

[22] Z. Wang , B. J. Wieder , J. Li , B. Yan , B. A. Bernevig , Phys. Rev. Lett. 2019, 123, 186401. 31763917 10.1103/PhysRevLett.123.186401

[23] R. Clarke , E. Marseglia , H. P. Hughes , Philos. Mag. B 1978, 38, 121.

[24] Y. Sun , S.‐C. Wu , M. N. Ali , C. Felser , B. H. Yan , Phys. Rev. B 2015, 92, 161107.

[25] Z. Wang , D. Gresch , A. A. Soluyanov , W. Xie , S. Kushwaha , X. Dai , M. Troyer , R. J. Cava , B. A. Bernevig , Phys. Rev. Lett. 2016, 117, 056805. 27517788 10.1103/PhysRevLett.117.056805

[26] K. Deng , G. L. Wan , P. Deng , K. N. Zhang , S. J. Ding , E. Wang , M. Z. Yan , H. Q. Huang , H. Y. Zhang , Z. L. Xu , J. Denlinger , A. Fedorov , H. T. Yang , W. H. Duan , H. Yao , Y. Wu , S. S. Fan , H. J. Zhang , X. Chen , S. Y. Zhou , Nat. Phys. 2016, 12, 1105.

[27] L. Huang , T. M. McCormick , M. Ochi , Z. Zhao , M.‐T. Suzuki , R. Arita , Y. Wu , D. Mou , H. Cao , J. Yan , N. Trivedi , A. Kaminski , Nat. Mater. 2016, 15, 1155. 27400386 10.1038/nmat4685

[28] J. Jiang , Z. K. Liu , Y. Sun , H. F. Yang , C. R. Rajamathi , Y. P. Qi , L. X. Yang , C. Chen , H. Peng , C.‐C. Hwang , S. Z. Sun , S.‐K. Mo , I. Vobornik , J. Fujii , S. S. P. Parkin , C. Felser , B. H. Yan , Y. L. Chen , Nat. Commun. 2017, 8, 13973. 28082746 10.1038/ncomms13973 PMC5241795

[29] A. Liang , J. Huang , S.i. Nie , Y. Ding , Q. Gao , C. Hu , S. He , Y. Zhang , C. Wang , B. Shen , J. Liu , P. Ai , L. Yu , X. Sun , W. Zhao , S. Lv , D. Liu , C. Li , Y. Zhang , Y. Hu , Y. Xu , L. Zhao , G. Liu , Z. Mao , X. Jia , F. Zhang , S. Zhang , F. Yang , Z. Wang , Q. Peng , et al., arXiv 2016, 1604, 01706.

[30] N. Xu , Z. J. Wang , A. P. Weber , A. Magrez , P. Bugnon , H. Berger , C. E. Matt , J. Z. Ma , B. B. Fu , B. Q. Lv , N. C. Plumb , M. Radovic , E. Pomjakushina , K. Conder , T. Qian , J. H. Dil , J. Mesot , H. Ding , M. Shi , arXiv 2016, 1604, 02116.

[31] A. Tamai , Q. S. Wu , I. Cucchi , F. Y. Bruno , S. Riccò , T. K. Kim , M. Hoesch , C. Barreteau , E. Giannini , C. Besnard , A. A. Soluyanov , F. Baumberger , Phys. Rev. X 2016, 6, 031021.

[32] M. Y. Zhang , Z. X. Wang , Y. N. Li , L. Y. Shi , D. Wu , T. Lin , S. J. Zhang , Y. Q. Liu , Q. M. Liu , J. Wang , T. Dong , N. L. Wang , Phys. Rev. X 2019, 9, 021036.

[33] C. Heikes , I.‐L. Liu , T. Metz , C. Eckberg , P. Neves , Y. Wu , L. Hung , P. Piccoli , H. B. Cao , J. Leao , J. Paglione , T. Yildirim , N. P. Butch , W. Ratcliff II , Phys. Rev. Mater. 2018, 2, 074202. 10.1103/physrevmaterials.2.074202 PMC11520192 39474140

[34] P. Li , J. Cui , J. Zhou , D. Guo , Z. Zhao , J. Yi , J. Fan , Z. Ji , X. Jing , F. Qu , C. Yang , L. Lu , J. Lin , Z. Liu , G. Liu , Adv. Mater. 2019, 31, 1904641. 10.1002/adma.201904641 31595592

[35] K. Zhang , C. Bao , Q. Gu , X. Ren , H. Zhang , K. Deng , Y. Wu , Y. Li , J. Feng , S. Zhou , Nat. Commun. 2016, 7, 13552. 27934874 10.1038/ncomms13552 PMC5155143

[36] L. Zhou , A. Zubair , Z. Wang , X. Zhang , F. Ouyang , K. Xu , W. Fang , K. Ueno , J. Li , T. Palacios , J. Kong , M. S. Dresselhaus , Adv. Mater. 2016, 28, 9526. 27620354 10.1002/adma.201602687

[37] S.‐Y. Chen , T. Goldstein , D. Venkataraman , A. Ramasubramaniam , J. Yan , Nano Lett. 2016, 16, 5852. 27517466 10.1021/acs.nanolett.6b02666

[38] K. Chen , Z. Chen , X. Wan , Z. Zheng , F. Xie , W. Chen , X. Gui , H. Chen , W. Xie , J. Xu , Adv. Mater. 2017, 29, 1700704. 10.1002/adma.201700704 28833622

[39] L. Zhou , S. X. Huang , Y. Tatsumi , L. J. Wu , H. H. Guo , Y.‐Q. Bie , K. Ueno , T. Yang , Y. M. Zhu , J. Kong , R. Saito , M. Dresselhaus , J. Am. Chem. Soc. 2017, 139, 8396. 28541698 10.1021/jacs.7b03445

[40] A. M. Zhang , X. L. Ma , C. L. Liu , R. Lou , Y. M. Wang , Q. H. Yu , Y. Y. Wang , T.‐L. Xia , S. C. Wang , L. Zhang , X. Q. Wang , C. F. Chen , Q. M. Zhang , Phys. Rev. B 2019, 100, 201107(R).

[41] Y. Huang , Y.‐H. Pan , R. Yang , L.‐H. Bao , L. Meng , H.‐L. Luo , Y.‐Q. Cai , G.‐D. Liu , W.‐J. Zhao , Z. Zhou , L.‐M. Wu , Z.‐L. Zhu , M. Huang , L.‐W. Liu , L. Liu , P. Cheng , K.‐H. Wu , S.‐B. Tian , C.‐Z. Gu , Y.‐G. Shi , Y.‐F. Guo , Z. G. Cheng , J.‐P. Hu , L. Zhao , G.‐H. Yang , E. Sutter , P. Sutter , Y.‐L. Wang , W. Ji , X.‐J. Zhou , et al., Nat. Commun. 2020, 11, 2453. 32415180 10.1038/s41467-020-16266-w PMC7228924

[42] J. M. Li , Y. K. Yao , L. H. Sun , X. Y. Shan , C. Wang , X. H. Lu , Chin. Phys. Lett. 2019, 36, 048201.

[43] R. Beams , L. G. Cançado , S. Krylyuk , I. Kalish , B. Kalanyan , A. K. Singh , K. Choudhary , A. Bruma , P. M. Vora , F. Tavazza , A. V. Davydov , S. J. Stranick , ACS Nano 2016, 10, 9626. 27704774 10.1021/acsnano.6b05127 PMC5542881

[44] Y. Cheon , S. Y. Lim , K. Kim , H. Cheong , ACS Nano 2021, 15, 2962. 33480685 10.1021/acsnano.0c09162

[45] Q. Song , H. Wang , X. Pan , X. Xu , Y. Wang , Y. Li , F. Song , X. Wan , Y. Ye , L. Dai , Sci. Rep. 2017, 7, 1758. 28496170 10.1038/s41598-017-01874-2 PMC5431984

[46] R. He , S. Z. Zhong , H. H. Kim , G. H. Ye , Z. P. Ye , L. Winford , D. McHaffie , I. Rilak , F. C. Chen , X. Luo , Y. P. Sun , A. W. Tsen , Phys. Rev. B 2018, 97, 041410.

[47] S. Paul , S. Karak , M. Mandal , A. Ram , S. Marik , R. P. Singh , S. Saha , Phys. Rev. B 2020, 102, 054103.

[48] D. Rhodes , D. A. Chenet , B. E. Janicek , C. Nyby , Y. Lin , W. Jin , D. Edelberg , E. Mannebach , N. Finney , A. Antony , T. Schiros , T. Klarr , A. Mazzoni , M. Chin , Y.‐C. Chiu , W. Zheng , Q. R. Zhang , F. Ernst , J. I. Dadap , X. Tong , J. Ma , R. Lou , S. Wang , T. Qian , H. Ding , R. M. Osgood Jr , D. W. Paley , A. M. Lindenberg , P. Y. Huang , A. N. Pasupathy , et al., Nano Lett. 2017, 17, 1616. 28145719 10.1021/acs.nanolett.6b04814

[49] M. W. Iqbal , K. Shahzad , R. Akbar , G. Hussain , Microelectron. Eng. 2020, 219, 111152.

[50] S. Das , K. Debnath , B. Chakraborty , A. Singh , S. Grover , D. V. S. Muthu , U. V. Waghmare , A. K. Sood , Nanotechnology 2021, 32, 045202. 33036010 10.1088/1361-6528/abbfd6

[51] Y. Yoo , Z. P. DeGregorio , Y. Su , S. J. Koester , J. E. Johns , Adv. Mater. 2017, 29, 1605461. 10.1002/adma.201605461 28221704

[52] C. Kim , I. Moon , D. Lee , M. S. Choi , F. Ahmed , S. Nam , Y. Cho , H.‐J. Shin , S. Park , W. J. Yoo , ACS Nano 2017, 11, 1588. 28088846 10.1021/acsnano.6b07159

[53] H.‐J. Kim , S.‐H. Kang , I. Hamada , Y.‐W. Son , Phys. Rev. B 2017, 95, 180101.

[54] G. Stan , C. V. Ciobanu , S. R. J. Likith , A. Rani , S. Zhang , C. A. Hacker , S. Krylyk , A. V. Davydov , ACS Appl. Mater. Interfaces 2020, 12, 18182. 32192325 10.1021/acsami.0c04339 PMC7425619

[55] J. Gao , B. Li , J. Tan , P. Chow , T. M. Lu , N. Koratkar , ACS Nano 2016, 10, 2628. 26808328 10.1021/acsnano.5b07677

[56] S. Tongay , J. Zhou , C. Ataca , J. Liu , J. S. Kang , T. S. Matthews , L. You , J. Li , J. C. Grossman , J. Wu , Nano Lett. 2013, 13, 2831. 23627637 10.1021/nl4011172

[57] H. Nan , Z. Wang , W. Wang , Z. Liang , Y. Lu , Q. Chen , D. He , P. Tan , F. Miao , X. Wang , J. Wang , ACS Nano 2014, 8, 5738. 24836121 10.1021/nn500532f

[58] P. L. Levesque , S. S. Sabri , C. M. Aguirre , J. Guillemette , M. Siaj , P. Desjardins , T. Szkopek , R. Martel , Nano Lett. 2010, 11, 132. 21141990 10.1021/nl103015w

