# Supplementary material for: Persistence of Monoclinic Crystal Structure in 3D Second‐Order Topological Insulator Candidate 1T′‐MoTe2 Thin Flake Without Structural Phase Transition
Source: Adv Sci (Weinh). 2021 Dec 19;9(5):2101532. doi: 10.1002/advs.202101532 (PMC8844473; doi:10.1002/advs.202101532)
Supplement: Supplementary file 1 — Supporting Information [file ADVS-9-2101532-s001.pdf]

WILEY-VCH

ADVANCED SCIENCE
Open Access

Supporting Information

for *Adv. Sci.*, DOI: 10.1002/advs.202101532

Persistence of Monoclinic Crystal Structure in Three-Dimensional Second-Order Topological Insulator Candidate 1T'-MoTe2 Thin Flake without Structural Phase transition

*Bo Su,# Yuan Huang,# Yan Hui Hou, Jiawei Li, Rong Yang, Yongchang Ma, Yang Yang, Guangyu Zhang, Xingjiang Zhou, Jianlin Luo, and Zhi-Guo Chen**

WILEY-VCH

© 2021 Wiley-VCH GmbH

Supporting Information

Persistence of Monoclinic Crystal Structure in Three-Dimensional Second-Order Topological Insulator Candidate 1*T'*-$MoTe_2$ Thin Flake without Structural Phase transition

Bo Su,[#] *Yuan Huang,*[#] *Yan Hui Hou, Jiawei Li, Rong Yang, Yongchang Ma, Yang Yang, Guangyu Zhang, Xingjiang Zhou, Jianlin Luo, and Zhi-Guo Chen**

B. Su, Prof. Y. Huang, Y. H. Hou, J. Li, Prof. R. Yang, Prof. Y. Yang, Prof. G. Zhang, Prof. X. Zhou, Prof. J. Luo, Prof. Z.-G. Chen
Beijing National Laboratory for Condensed Matter Physics
Institute of Physics, Chinese Academy of Sciences
Beijing 100190, China
Email: zgchen@iphy.ac.cn

B. Su, J. Li, Prof. G. Zhang, Prof. X. Zhou, Prof. J. Luo, Prof. Z.-G. Chen
School of Physical Sciences
University of Chinese Academy of Sciences
Beijing 100190, China

Y. H. Hou, Prof. Y. Ma
School of Materials Science and Engineering
Tianjin University of Technology
Tianjin 300384, China

Prof. Y. Huang, Prof. R. Yang, Prof. Y. Yang, Prof. G. Zhang, Prof. X. Zhou, Prof. J. Luo, Prof. Z.-G. Chen
Songshan Lake Materials Laboratory
Dongguan, Guangdong, 523808, China

Prof. G. Zhang, Prof. X. Zhou, Prof. J. Luo
Collaborative Innovation Center of Quantum Matter
Beijing, China

CONTENT

Figure S1. Raman spectra of the $MoTe_2$ bulk crystal measured with the orthorhombic T_d structure at 80 K and with a monoclinic 1*T'* structure at 300 K, respectively

Figure S2. Temperature dependence of the Raman spectra of the $MoTe_2$ thin flakes with the thickness varying from ~ 150.0 nm to ~ 13.8 nm

Figure S3. Raman spectra of the $MoTe_2$ thin flakes with two different thicknesses and Raman spectra of the $MoTe_2$ flakes measured at four different temperatures

SUPPLEMENTAL FIGURES

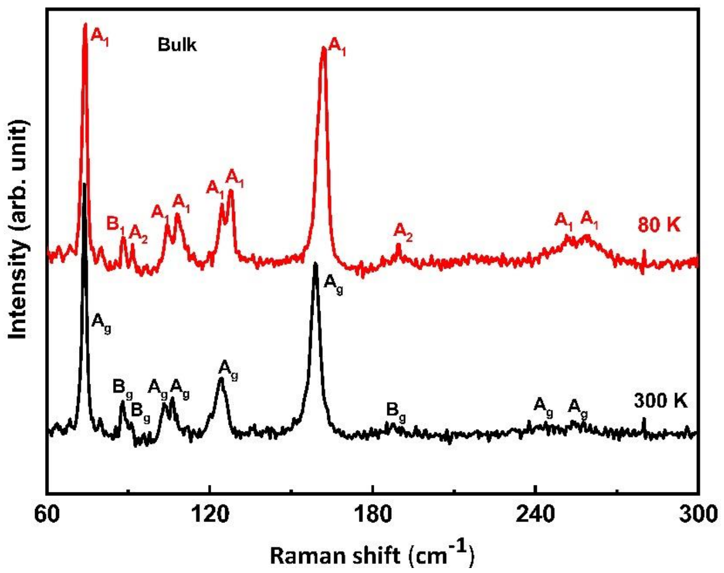

Figure S1. Raman spectra of the $MoTe_2$ bulk crystal measured with the orthorhombic T_d structure at 80 K and with a monoclinic 1*T'* structure at 300 K, respectively.

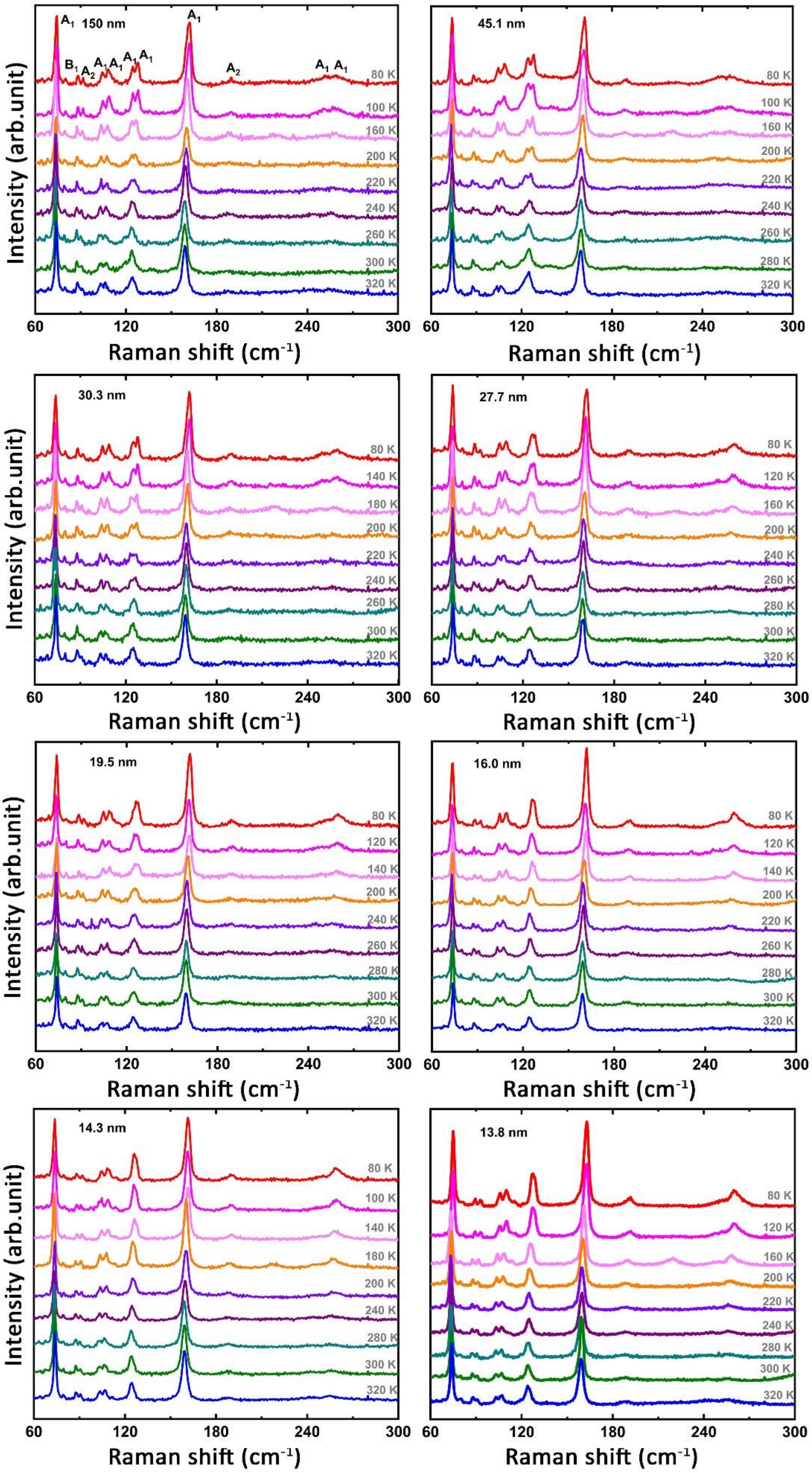

Figure S2. Temperature dependence of the Raman spectra of the $MoTe_2$ thin flakes with the thickness varying from ~ 150.0 nm to ~ 13.8 nm.

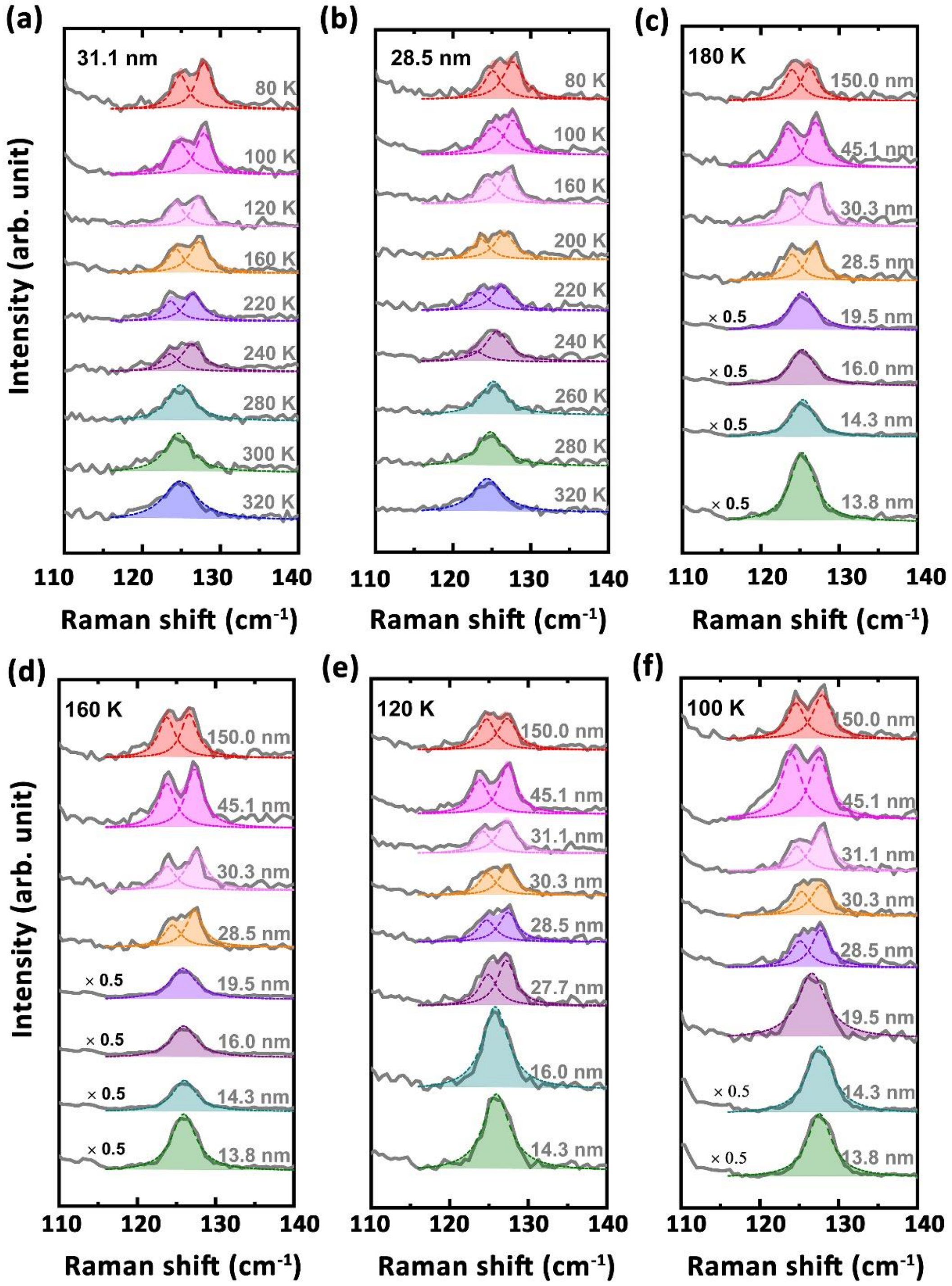

Figure S3. a), b) Raman spectra of the $MoTe_2$ thin flakes with the thicknesses of ~ 31.1 nm and ~ 28.5 nm. c)-f) Raman spectra of the $MoTe_2$ thin flakes measured at T = 180 K, 160 K, 120 K and 100 K, respectively. The Raman spectra labelled with "× 0.5" in (d) and (f) are shown with the half of the intensities.
